# STArS (STrain-Amplicon-Seq), a targeted nanopore sequencing workflow for SARS-CoV-2 diagnostics and genotyping

**DOI:** 10.1093/biomethods/bpac020

**Published:** 2022-08-25

**Authors:** Simone Maestri, Valentina Grosso, Massimiliano Alfano, Denise Lavezzari, Chiara Piubelli, Zeno Bisoffi, Marzia Rossato, Massimo Delledonne

**Affiliations:** Department of Biotechnology, University of Verona, 37134 Verona, Italy; Center for Genomic Science of IIT@SEMM, Fondazione Istituto Italiano di Tecnologia, 20139 Milano, Italy; Department of Biotechnology, University of Verona, 37134 Verona, Italy; Department of Biotechnology, University of Verona, 37134 Verona, Italy; Department of Biotechnology, University of Verona, 37134 Verona, Italy; Department of Infectious, Tropical Diseases and Microbiology, IRCCS Sacro Cuore Don Calabria Hospital, 37024 Verona, Italy; Department of Infectious, Tropical Diseases and Microbiology, IRCCS Sacro Cuore Don Calabria Hospital, 37024 Verona, Italy; Department of Diagnostics and Public Health, University of Verona, 37134, Verona, Italy; Department of Biotechnology, University of Verona, 37134 Verona, Italy; Genartis srl, 37126 Verona, Italy; Department of Biotechnology, University of Verona, 37134 Verona, Italy; Genartis srl, 37126 Verona, Italy

## Abstract

Diagnostic tests based on reverse transcription–quantitative polymerase chain reaction (RT–qPCR) are the gold standard approach to detect severe acute respiratory syndrome coronavirus 2 (SARS-CoV-2) infection from clinical specimens. However, unless specifically optimized, this method is usually unable to recognize the specific viral strain responsible of coronavirus disease 2019, a crucial information that is proving increasingly important in relation to virus spread and treatment effectiveness. Even if some RT–qPCR commercial assays are currently being developed for the detection of viral strains, they focus only on single/few genetic variants that may not be sufficient to uniquely identify a specific strain. Therefore, genome sequencing approaches remain the most comprehensive solution for virus genotyping and to recognize viral strains, but their application is much less widespread due to higher costs. Starting from the well-established ARTIC protocol coupled to nanopore sequencing, in this work, we developed STArS (STrain-Amplicon-Seq), a cost/time-effective sequencing-based workflow for both SARS-CoV-2 diagnostics and genotyping. A set of 10 amplicons was initially selected from the ARTIC tiling panel, to cover: (i) all the main biologically relevant genetic variants located on the Spike gene; (ii) a minimal set of variants to uniquely identify the currently circulating strains; (iii) genomic sites usually amplified by RT–qPCR method to identify SARS-CoV-2 presence. PCR-amplified clinical samples (both positive and negative for SARS-CoV-2 presence) were pooled together with a serially diluted exogenous amplicon at known concentration and sequenced on a MinION device. Thanks to a scoring rule, STArS had the capability to accurately classify positive samples in agreement with RT–qPCR results, both at the qualitative and quantitative level. Moreover, the method allowed to effectively genotype strain-specific variants and thus also return the phylogenetic classification of SARS-CoV-2-postive samples. Thanks to the reduced turnaround time and costs, the proposed approach represents a step towards simplifying the clinical application of sequencing for viral genotyping, hopefully aiding in combatting the global pandemic.

## Introduction

Since the emergence of severe acute respiratory syndrome coronavirus 2 (SARS-CoV-2) in late 2019, cases of coronavirus disease 2019 (COVID-19) have quickly emerged around the world, causing millions of deaths [1, 2].

Reverse transcription–quantitative polymerase chain recation (RT–qPCR) assay is the gold standard assay for SARS-CoV-2 detection and the standard diagnostic test for COVID-19 in clinical practice [3]. Despite being very specific, time and cost-effective, the RT–qPCR method exhibits a high false-negative rate because it detects only a few genomic sites that may not be amplified in low titre samples or when mutations occur [4]. A novel method called LamPORE, based on LAMP amplification coupled with nanopore sequencing was recently proposed for screening a large number of samples for the presence of SARS-CoV-2 [1]. Although very useful for fast and high-throughput screening, this method showed a higher limit of detection compared to RT–qPCR, and it does not provide information about the viral load [1, 5, 6]. Both RT–qPCR and LamPORE do not provide sequence information of the amplified gene fragment and are therefore unable to recognize the specific viral strain responsible of infection.

The emergence of novel circulating strains of SARS-CoV-2 has raised significant concerns on the ability to confine viral spread and on the efficacy of treatments/vaccines [7, 8]. The European Center for Disease Prevention and Control (ECDC) declared that a tight monitoring of variants of concern (VOCs) in all countries is key to control virus evolution and inform appropriate decisions on vaccine composition and outbreak analyses [9]. Analysis of (known and unknown) SARS-CoV-2 variants should be routinely conducted next to standard diagnostic testing and exploited to address more appropriate treatments, patient management and public health measures [9].

However, there are several challenges to the analyses of VOCs, particularly in real time. Current methods for SARS-CoV-2 testing, namely detection of viral RNA by RT–qPCR or antigen-based testing—in their standard formulation—are unable to recognize the specific viral strain responsible of infection. Most importantly, some VOCs show the ability to elude these diagnostic tests, as strain-specific genetic variants can occur on the assay-recognition sites [10]. Some companies are currently developing PCR/antigenic-based tests for the recognition of some selected (1–2) most common VOCs. However, this effort requires extensive testing and assay optimization to exclude cross-reactivity. Moreover, these assays will be far to be strain-specific and complete, as single PCR/antigen-based assays cannot efficiently analyse more than two to three different variation sites. In contrast, each VOC is characterized by numerous genetic variants (Omicron variant has 30 mutations [11]) and different VOCs frequently share some variations. Only analysing a big number of genetic sites would allow the unambiguous identification of a specific VOC, therefore PCR/antigen-based results will always require orthogonal validation based on genome sequencing. For these reasons, ECDC declares that the only way to identify and characterize new variants and unambiguously type existing variants is using whole-genome sequencing (WGS) of SARS-CoV-2 [9]. However, WGS is usually not implemented in diagnostic laboratories, because it requires consistently longer times (days versus hours of standard testing), that are not compatible with public health response (e.g. contact tracing). In addition, WGS is still a relatively expensive method as compared to PCR-based testing, it requires high investment in equipment, extensive training and it is usually less standardized, especially in the downstream data analyses. Finally, WGS is usually not quantitative and does not allow to infer the viral load present in samples, a critical feature when considering environmental monitoring.

Today, sequencing of SARS-CoV-2 genome is the only available approach to comprehensively perform viral genome genotyping, for the identification of the viral strain responsible of a COVID-19 infection. In contrast to other methods, such as RT–qPCR, sequencing does not rely on the analysis of a pre-defined set of known SNPs, nor it requires to be progressively optimized for their analysis. Sequencing of SARS-CoV-2 genomes was indeed exploited by different consortia to complement routine diagnostic testing, and to identify the phylogenetic structure of disease outbreaks [3, 12, 13]. Viral genome sequencing implemented by public health agencies proved the concept that data integration and sharing provide a valuable means to improve the management of the emerging disease [14, 15]. Moreover, this global pandemic strongly highlighted the importance of quick monitoring of disease outbreaks to inform quarantine decisions or contact tracing [1]. Recently, the emergence of novel variants in SARS-CoV-2 genome raised great concern in the scientific community, since they may lead to more contagious and lethal strains [16–20]. In particular, variants occurring in the Spike protein may compromise the efficacy of available vaccines, thus requiring sequencing efforts to track circulating strains [21]. Among those, strains first documented in the UK, Brazil, South Africa and India quickly became the dominant lineages in delimited areas, suggesting that they may be associated to an increased transmissibility [11, 19, 22, 23]. In particular, the Omicron variant is the most divergent strain sequenced so far during the pandemic, raising concerns that it may be linked to greater transmissibility compared to the Delta variant, lower vaccine efficiency and an increased risk of reinfection [11].

A variety of approaches have been used to sequence SARS-CoV-2 genomes including metagenomics [2], direct RNA sequencing [24, 25], targeted sequencing based on oligonucleotide-probe hybridization [26] or tiling multiplex amplicons [3, 4, 27–31]. Currently, PCR-based target enrichment is the most frequently used method for large-scale monitoring, thanks to its high-specificity and sensitivity across a wide range of viral input titres [28, 29]. In contrast to other methods that enable the analysis only of samples for high viral genome concentration, the amplicon-based viral sequencing can be applied also to specimens with low and very low titres, as well as to low quality samples [2, 32]. The first amplification scheme for SARS-CoV-2 sequencing was developed by the ARTIC network at the end of January 2020 [33], and consisted of 98 overlapping amplicons. The protocol was progressively improved, with the replacement of low-performance amplicons and it is now at the fourth version [29]. Genomic fragments amplified using the ARTIC panel can be sequenced either using short-reads (Illumina, MGI, Ion Torrent) or long-reads (Oxford Nanopore Technologies [ONT[, Pacific Biosciences). Thanks to the remarkable sequencing accuracy, Illumina sequencing represents the world-wide gold standard for sequencing-based genotyping, also for SARS-CoV-2 [3, 32]. The majority of data available in GISAID [34], an initiative promoting the rapid sharing of data from all influenza viruses, were indeed produced with Illumina platforms. The Illumina COVIDSeq protocol was recently approved by FDA Emergency Use Authorization (EUA) and it couples multiplex-PCR and sequencing of samples for high-throughput detection of SARS-CoV-2 [35, 36]. However, short-read platforms suffer of long turnaround time and the need for a dedicated infrastructure, strongly limiting flexibility [37, 38], an aspect that clashes with the need of fast decision making in pandemic control and patient management. The same aspects are critical also in those areas of the world that do not have sequencing facilities. Moreover, such protocols are optimized for large sample batches, but may not be cost-effective for small batches [35]. In contrast, ONT sequencing devices are highly portable, have minimal infrastructure requirements and can produce data in real time. The potential of ONT sequencing has been already reported for the Ebola and Zika outbreaks [39, 40] and recently the reliability of SARS-CoV-2 genotyping based on ONT-sequencing coupled to the ARTIC protocol was demonstrated [3]. Bull *et al*. indeed reported that the accurate detection of single-nucleotide variant can be achieved with a coverage of 60× [3, 41], thus allowing an efficient characterization of SARS-CoV-2 strains underlying the infection [3]. Moreover, Wang *et al*. [4] demonstrated that, after 10 min of sequencing, high-copy samples already produced enough sequencing data for diagnosis, and by extending the sequencing time to 1 h also low-copy samples could be identified.

In the present study, we developed a cost-efficient workflow (STArS) exploiting a subset of highly informative ARTIC amplicons and ONT targeted sequencing, that enables the rapid detection of SARS-CoV-2 presence in clinical specimens as well as its simultaneous genotyping. The assay can be performed in short time (comparable to traditional RT–qPCR) and at similar costs. As such, STArS translates a protocol mainly used in the research settings in a simple workflow for SARS-CoV-2 diagnosis/genotyping and potentially applicable in the clinic after the required certifications.

## Materials and methods

### Clinical samples

Nasopharyngeal swabs were collected from COVID-19 patients diagnosed at the Department of Infectious, Tropical Diseases and Microbiology of the IRCCS Sacro Cuore Don Calabria Hospital, qualified for SARS-CoV-2 molecular diagnosis by the regional reference laboratory (Department of Microbiology, University Hospital of Padua). After collection, swabs were placed in a viral transport medium, analysed by the molecular diagnostic method (described in the following paragraph) and subsequently stored at −80°C.

### RNA extraction and RT–qPCR analysis

RNA was isolated using QIAamp viral RNA mini kit (Qiagen) by spin-column procedure according to the manufacturer’s instructions. Samples were eluted twice in 50 µl of dH_2_O and concentrations and purity were checked on a Nanodrop instrument. A volume of 5 µl of extracted RNA was used to perform the RT–qPCR detection using the TaqMan™ 2019-nCoV Assay Kit v1 (ThermoFisher) and the 2019-nCoV CDC qPCR Probe Assay [42]. In both cases, the cycling protocol recommended by each supplier was used in the QuantStudio3 Real Time PCR System (Thermo Fisher Scientific), using the samples obtained from the five SARS-CoV-2 positive individuals, the positive control (PC) provided by each kit together with SARS-CoV-2-free RNA, which was used as a negative control. Data were analysed using the relative quantification template in the instrument software.

### Reverse transcription and amplification of the SARS-CoV-2 genome

RNA samples were directly used for first-strand synthesis using the SuperScript IV kit (Thermo Fisher) and random hexamers. In brief, 5 μl RNA were mixed with 1 μl of random hexamers (50 μM, NEB) and 1 μl of dNTPs (10 mM, Invitrogen). The mixture was incubated for 5 minutes at 65°C, followed by 1 min on ice. Subsequently, 8 μl of enzyme mix containing 4 μl Super Script IV buffer (NEB), 1 μl 0.1 M DTT (NEB), 1 μl RNase OUT (Invitrogen) and 1 μl SuperScript IV were added to the samples. The reactions were placed in a thermocycler and incubated 10 min at 23°C, followed by 10 min at 52°C and 1 min at 80°C before cooling to 4°C.

Target amplicons were amplified in a 25 µl reaction system with 5 µl of cDNA, 3.6 µl of primers pool (10 mM total) and 12.5 µl of Q5 hot start high fidelity Master Mix (NEB). Each cDNA sample was amplified in replicates using the following programs: 98°C for 30 s and 30 cycles at 98°C for 10 s and 65°C for 5 min. The product was purified with 1× Ampure beads. PC samples were generated with the same protocol but using only primers for amplicon 96. The quantity of PC amplicons was measured with the Qubit 2.0 fluorometer using the dsDNA HS Assay Kit (Thermo Fisher Scientific, USA), and the size was assessed by electrophoresis using a Tapestation device (Agilent). PC dilutions were quantified by RT–qPCR as described above, aliquoted and stored at −20°C in Ethylenediamine Tetraacetic Acid; buffered solution (Tris-EDTA) buffer, avoiding freeze/thaw cycles PC samples were stable for more than 1 year without needing to repeat quantification.

### ONT sequencing

Library preparation for the ONT sequencing was performed using the Ligation Sequencing kit SQK-LSK109 and Native Barcoding kit EXP-NBD104 (ONT), according to the manufacturer’s instructions with minor modifications. Briefly, 5 µl of PCR amplicons were end-repaired and dA-tailed using an UltraII End Prep Reaction Module (NEB, USA) followed by ligation of native barcodes using the NEBNext UltraII Ligation module (NEB, USA). After pooling barcoded amplicons, libraries were purified using Ampure XP beads (Beckman Coulter) followed by adapters ligation with the NEBNext UltraII Ligation module. Library clean-up was performed using Ampure XP beads and short fragment buffer and then eluted in 15 μl of ONT’s elution buffer. The library for Experiment 1 was loaded onto an R9.4.1 flow cell (FLO-MIN106) and sequenced on a MinION Mk1B device for 3 h. The library for Experiment 2 was loaded onto an R9.4.1 Flongle flow-cell (FLO-FLG001) and sequenced on a MinION Mk1B device for 3 h. ONT MinKNOW software (version 19.12.2) was used to collect raw data and perform live basecalling (Guppy v3.4, fast mode).

The RAMPART tool (Read Assignment, Mapping and Phylogenetic Analysis in Real Time) developed by the ARTIC network (https://github.com/artic-network/rampart) was used to visualize genome coverage in real time and reference matching for each barcode.

### Analysis of ONT data

ONT reads were processed according to the ARTIC-nCOV-bioinformatics-SOP-v1.1.0 document [43].

In particular, raw fast5 files were base-called using Guppy v4.0.11 with high-accuracy algorithm with ‘guppy_basecaller -c dna_r9.4.1_450bps_hac.cfg -i/path/to/reads -r -s run_name’. Reads were then demultiplexed with Guppy v4.0.11, requiring the presence of indexes at both ends of the reads with ‘guppy_barcoder –require_barcodes_both_ends -i run_name -s output_directory –arrangements_files “barcode_arrs_nb12.cfg barcode_arrs_nb24.cfg’. Reads from each sample were then filtered keeping only reads with quality >7 and length in the 400–700 bp range with NanoFilt [44]. The filtered reads from each sample were then aligned to the reference genome (accession MN908947) with Minimap2 [45], keeping up to 200 reads from each strand. Portions of reads corresponding to PCR primers coordinates were then masked with soft-clipping, and variants were then called with Nanopolish variants (https://github.com/jts/nanopolish). Coordinates of the reference genome with coverage per strand <20 or with low-quality variants were used to build a mask. Finally, consensus sequences were generated with bcftools consensus [46]. All these steps were run with instruction ‘artic minion –normalise 200 –threads 8 –scheme-directory ∼/artic-ncov2019/primer_schemes –read-file run_name_barcode_i.fastq –fast5-directory path_to_fast5 –sequencing-summary path_to_sequencing_summary.txt nCoV-2019/V3 samplename’. Bedtools coverage v2.29.1 [47] was used to calculate the number of reads mapped in the non-overlapping portion of each amplicon and R with ggplot2 [48] was used to generate coverage plots. Scripts for running the whole pipeline are reported in https://github.com/MaestSi/Covid19_ONT_Artic repository.

The developed scoring system, adapted from [4], assigns a score to each amplicon and then the scores are combined to obtain a final score for the sample, for classifying it as positive to SARS-CoV-2, inconclusive or negative. The score for each amplicon is calculated by comparing the number of reads generated from it to the number of reads from the same amplicon assigned to the NTC sample. If this ratio is greater than 10, the amplicon is assigned a score of 1; if it is comprised between 3 and 10, the amplicon is assigned a score of 0.4; if it is lower than 3, the amplicon is assigned a score of 0. The final score for the sample is then obtained by summing up the scores of all amplicons. If the final score for the sample is greater than or equal to 2, then the sample is classified as positive; if it is lower than or equal to 1, the sample is classified as negative; otherwise, if the score is comprised between 1 and 2, the test is inconclusive. Compared to [4], all reads mapped to SARS-CoV-2 genome were retained and thresholds were adjusted to account for the different number of amplicons in the two experimental designs.

The vcf files produced for each sample were first intersected with a bed file containing the genomic coordinates of the variants of interest and were then compared with the list of known mutations characterizing strains of interest. The final consensus sequences were also uploaded in Nextclade [49], to obtain strain identification. This software is based on an empirical distance metric between query sequence and reference nodes which excludes from the computed distance all regions missing in the query sequence, making it suitable to work also with partial genomes.

## Results

### Generation of ONT sequencing data using STArS

To allow the simultaneous detection of SARS-CoV-2 and its genotyping, STArS workflow combines nanopore sequencing with one step RT–PCR of 10 regions of the viral genome. At this aim, 10 primer pairs were identified out of the 98 amplicons utilized by the ARTIC amplicon-sequencing method (Table 1). Specifically, two primer sets amplifying genomic sequences commonly detected by RT–qPCR assays, and eight primer sets amplifying genomic regions comprising a set of variant sites useful to recognize the main SARS-CoV-2 strains circulating at the end of May 2021, according to Pango Lineage [50]. As such, the assay setup allows to focus sequencing efforts on genomic regions which allow to discriminate among multiple strains, namely the ‘Alpha (UK lineage of concern)’ B.1.1.7, ‘Gamma (Brazilian lineage)’ P.1 or B.1.1.28.1, ‘Beta (South African lineage of concern)’ B.1.351, ‘UK lineage’ B.1.258, and ‘Eta lineage’ B.1.525 (Table 1). Although the assay was developed before the emergence of ‘Delta’ B.1.617.2 and ‘Omicron’ XE strains, the selected amplicons cover also unique variants featuring these strains, and they are thus expected to enable their identification (Table 1).

**Table 1.** ARTIC amplicons selected for STArS protocol

ARTIC amplicon	Genomic coordinates	SARS-CoV-2 Gene	Common mutations
51	15 171–15 560	ORF1b	− (detection amplicon)
71	21 357–21 743	ORF1b/S	L18F, T19R, T19I, T20N, L24S, del25/27, P26S
72	21 658–22 038	S	Q52R, del21765:6, D80A, D138Y, G142D, del21991:3
73	21 961–22 346	S	E156G, del157/158, R190S, V231G, D215G
75	22 516–22 903	S	G339D, S371F, S373P, S375F, T376A, D405N, R408S, K417T, K417N, N439K, N440K
76	22 797–23 214	S	L452R, S477N, T478K, E484K, E484A, Q493R, Q498R, N501Y, Y505H
77	23 122–23 522	S	A570D, D614G
78	23 443–23 847	S	H655Y, Q677H, N679K, P681H, P681R, A701V, T716I
81	24 391–24 789	S	D950N, Q954H, N969K, S982A, T1027I
93	28 081–28 464	ORF8/N	Y73C, S84L, E92K, del119/120, del31/33, D3L, del : 28278:3 (detection amplicon)

ARTIC amplicons selected for identifying and genotyping SARS-CoV-2 virus are reported, together with genomic positions and annotated gene.

The STArS assay was first applied on 11 clinical samples from nasopharyngeal swabs, presenting with variable viral load based on a standard RT–qPCR diagnostic assay, namely Cycle threshold (Ct), from 26.4 to 40.5 (Table 2). According to previous results, such Ct range corresponded to viral loads ranging from 1 to approximately 10 000 viral copies in the input sample utilized in the reaction. Possible contamination was monitored by amplifying a negative control sample (NTC, No Template Control) in parallel to clinical samples. STArS-selected amplicons were generated by maximizing the starting cDNA volume and were not purified or quantified prior to sequencing, as described in the materials and methods section. Barcoded ONT sequencing libraries were generated from equal amplicon reaction volumes, in order to maintain viral load ratios. According to the STArS workflow and the lowest sample Ct, a minimum of 10^7^–10^8^ single-amplicon copies was supposed to be sequenced for each clinical sample, when considering either 100% or 10% RT–PCR reaction efficiency, respectively [51, 52].

**Table 2.** SARS-CoV-2 RT–qPCR Ct values of clinical samples

Sequencing experiment	Sample ID	Ct N gene	Date of collection
Run 1	18	28.1	9 February 2021
	326	30.6	8 February 2021
	128	31.6	9 February 2021
	241	33.9	9 February 2021
	331	34.3	8 February 2021
	325	30.6	8 February 2021
	282	38.4	9 February 2021
	172	40.5	5 February 2021
	41	33.4	28 January 2021
	123	33.2	11 February 2021
	80	27.2	11 February 2021
Run 2	08	25.0	20 February 2021
	09	30.0	20 February 2021
	10	ND	20 February 2021

For each sample, the cycle threshold (Ct) values, as detected on N gene is reported. ND: not detectable.

In parallel, ONT sequencing libraries were generated from a PC sample that was used to monitor data production and the lower detection limit of STArS assay. PC was generated from a previously sequenced SARS-CoV-2 positive sample [32] by amplifying amplicon 96, encompassing part of N2 gene, not assayed by STArS in the tested clinical samples. The amplified product was 5-fold serially diluted 1:100, down to a concentration corresponding to 10^3^ copies per reaction, namely four orders of magnitude lower than the amplicon amount expected from the least concentrated clinical sample. All dilutions were quantified by RT–qPCR prior to library preparation, to ensure appropriate dilution and the detection level fell in the expected range, with the lowest input of 10^3^ copies detected at Ct 30 (Table 3). In parallel, the PC amplicon was also further diluted to a concentration corresponding to 10 copies per reaction and it was subjected to the entire STArS workflow, starting from amplification.

**Table 3.** RT–qPCR and sequencing results of PCs

Sequencing experiment	Sample ID	Ct	Sample barcode	Num. PASS reads	Num. PASS reads mapped to SARS-CoV-2 genome	Mean PASS filtered read length (SD)
Run 1	Ampl_96_10_copies_amplified_rep1	5.0	BC13	10 685	10 668	511 (24)
	Ampl_96_10_copies_amplified_rep2	5.0	BC14	7707	7696	511 (23)
	Ampl_96_10^9_copies_rep1	11.3	BC21	1809	1799	508 (13)
	Ampl_96_10^9_copies_rep2	11.3	BC22	1950	1943	508 (12)
	Ampl_96_10^7_copies_rep1	17.3	BC15	48	44	520 (42)
	Ampl_96_10^7_copies_rep2	17.3	BC16	22	22	503 (23)
	Ampl_96_10^5_copies_rep1	23.7	BC17	9	9	520 (37)
	Ampl_96_10^5_copies_rep2	23.7	BC18	6	5	546 (80)
	Ampl_96_10^3_copies_rep1	30.8	BC19	8	8	516 (24)
	Ampl_96_10^3_copies_rep2	30.8	BC20	7	7	534 (72)
Run 2	Ampl_96_10_copies_amplified	5.0	BC12	434	429	502 (25)

For each sample, the Ct, the length, the number of reads and number of reads mapped to SARS-CoV-2 genome are reported.

Clinical samples, NTC, and PC were sequenced in the same ONT sequencing run (Run 1) for 20 h, generating a total of 1,265,477 reads. Of those, 644,914 reads (50.9%) were retained after filtering based on quality and length and could be demultiplexed with stringent parameters.

### STArS limit of detection

**Figure 1:**
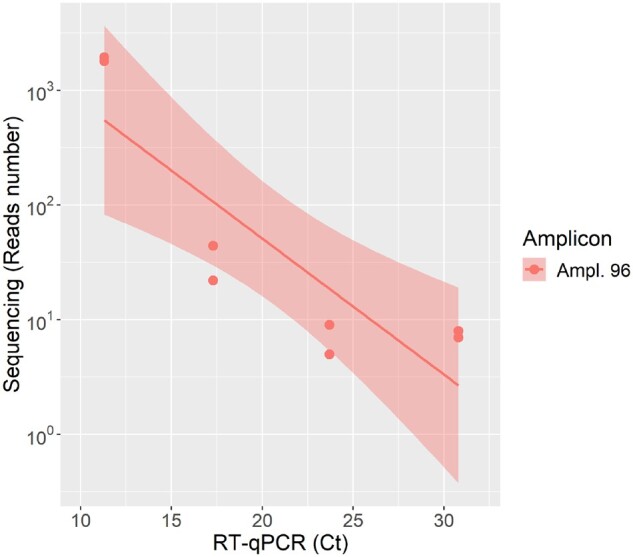
Correlation between sequencing and RT–qPCR results for PC. The number of reads assigned to each dilution of amplicon 96 of the PC and their Ct is reported. Linear regression lines and confidence intervals are also shown.

To determine the limit of detection of the STArS protocol, the reads generated from serial dilutions of the PC, corresponding to variable numbers of amplicon copies in library preparation were initially analysed. Increasing amounts of sequencing reads were assigned to each PC dilution (Table 3). A statistically significant correlation was observed between the logarithmic number of generated sequencing reads, ranging from 5 to 10,668, and the Ct of the starting amplicon copy number (Pearson *r* = −0.864, *t* test, *P* = 0.005) (Fig. 1). When sequencing libraries were directly generated from 10^5^ or 10^3^ amplicons, the number of reads assigned to SARS-CoV-2 genome was neither consistently above the NTC sample nor proportional to the theoretical input amplicon copies. Conversely, when introducing 10^7^ amplicon copies in library preparation, the number of reads assigned to PC samples, and attributable to SARS-CoV-2, was consistently higher compared to the NTC sample (Table 3). This was therefore identified as STArS limit of detection.

In order to classify samples as positive for SARS-CoV-2, a scoring system adapted from Wang et al. 2020 [4], that considers the coverage depth of all target regions in comparison to NTC coverage, was developed, according to the formula reported in the materials and methods section. STArS score ranges from 0 to 10 and samples are classified as positive when a score above 2 is assigned.

### SARS-CoV-2 detection by STArS

**Figure 2:**
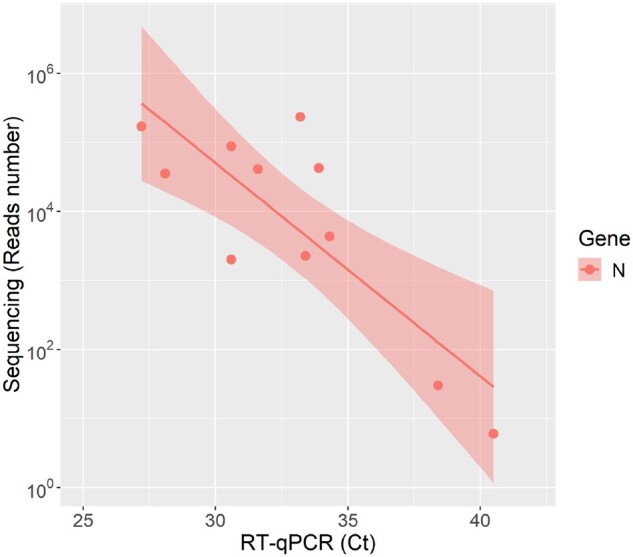
Correlation between sequencing and RT–qPCR results. The number of reads assigned to each sample and their Ct on N gene is reported. Linear regression lines and confidence intervals are also shown.

**Figure 3:**
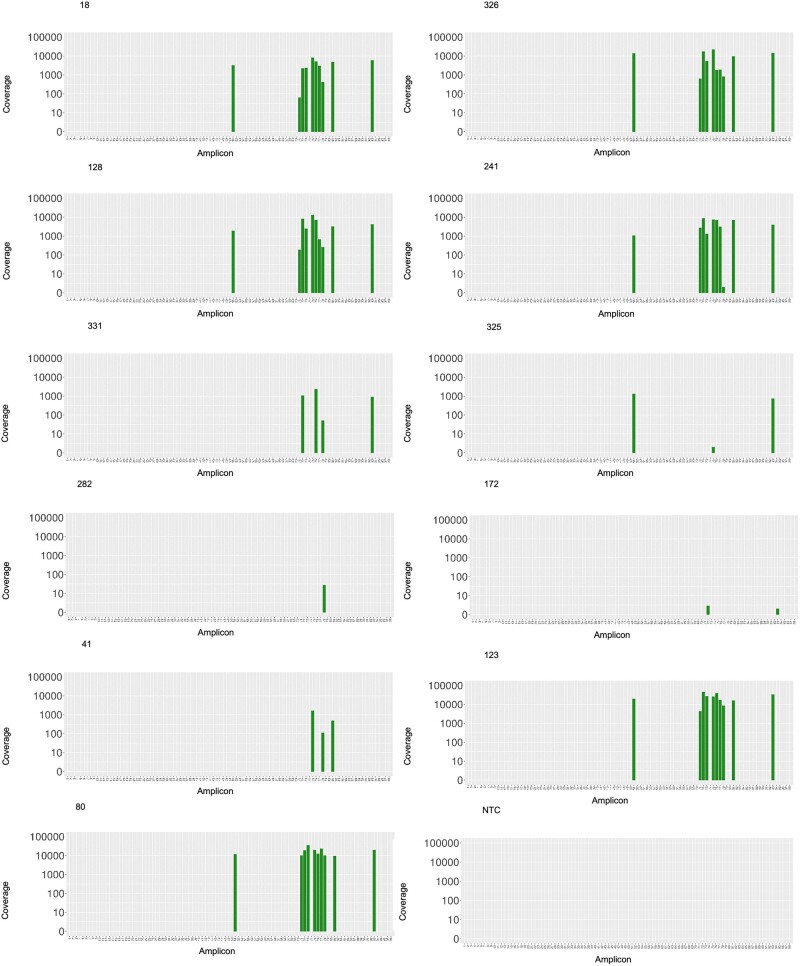
SARS-CoV-2 genome sequencing coverage for Run 1. For each sample, the number of reads mapped to each amplicon is reported in log10 scale.

After sequencing with the STArS protocol (Run 1), each clinical sample generated on average 56,529 reads, of which 99.8% were properly mapped to SARS-CoV-2 genome (Table 4). The NTC sample yielded 717 reads, but only two could be assigned to the SARS-CoV-2 genome. The logarithmic number of reads assigned to each sample was negatively correlated to the Ct value of N gene, with a significant statistical correlation (Pearson *r* = −0.818, *t* test, *P*  = 0.002) (Fig. 2). Consistently with previous reports [32], in samples with medium/high-viral loads (Ct < 30) all amplicons were detected while in samples with low viral titre (Ct > 30) amplicons were randomly missing (Fig. 3). Notably, also samples with the lowest viral loads, Ct 38.4 and 40.5 (3 and 1 theoretical starting viral copies), generated a number of reads above the NTC threshold, namely 30 and 6 reads mapped to the SARS-CoV-2 genome, respectively.

**Table 4.** Sequencing results of clinical samples

Sequencing experiment	Sample ID	Sample barcode	Num. PASS reads	Num. PASS reads mapped to SARS-CoV-2 genome	Mean PASS filtered read length (SD)	Score	Outcome
Run 1	18	BC01	35 450	35 369	509 (15)	10	+
	326	BC02	87 785	87 675	504 (12)	10	+
	128	BC03	41 116	41 092	505 (17)	10	+
	241	BC04	42 388	42 330	508 (15)	10	+
	331	BC05	4396	4341	515 (21)	4	+
	325	BC06	2096	2031	504 (18)	7	+
	282	BC07	50	30	520 (53)	3	+
	172	BC08	55	6	526 (78)	3	+
	41	BC09	2448	2268	509 (26)	4	+
	123	BC10	235 674	235 483	508 (15)	10	+
	80	BC11	170 356	170 018	502 (18)	10	+
	NTC	BC12	717	2	490 (75)	NA	NA
Run 2	08	BC08	456	448	503 (16)	9	+
	09	BC09	13	13	501 (15)	3	+
	10	BC10	1	0	478 (NA)	0	−
	NTC	BC11	0	0	−	NA	NA

For each sample, the number and length of reads are reported. Moreover, the total Score is reported, together with the predicted outcome. ‘+’: positive classification; ‘−’: negative classification; ‘?’: inconclusive test. NTC: no template control.

All clinical samples were classified as positive for SARS-CoV-2 based on our scoring rule, in agreement with RT–qPCR analysis (Table 4).

### SARS-CoV-2 genotyping by STArS

**Figure 4:**
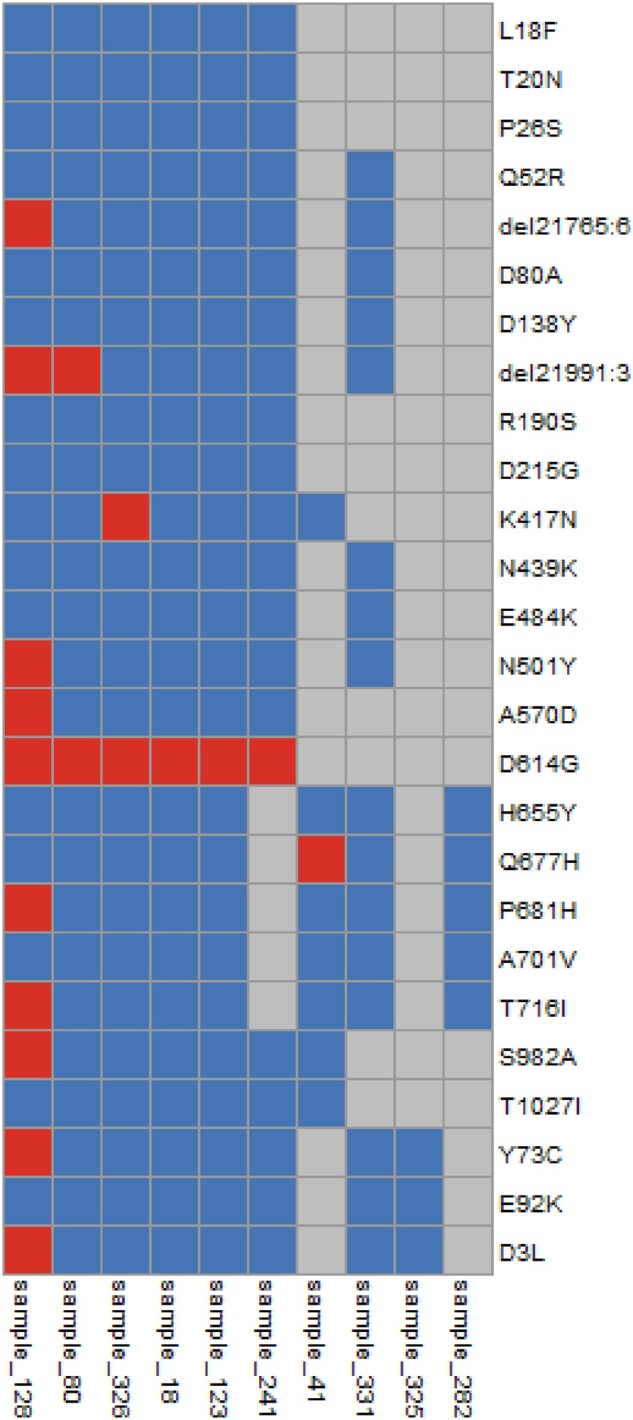
Identification of SARS-CoV-2 strains using STArS. The heatmap represents the distribution of a subset of mutations of interest (rows) across the samples (columns). Blue stands for reference genotype, red stands for variant genotype, while grey stands for not genotypable position. Sample ‘172’ was excluded from the heatmap, since no position was genotypable.

In order to identify the SARS-CoV-2 strain, variant calling was performed on the sequenced amplicons. The set of identified variants in each sample was intersected with genomic coordinates associated with variants of circulating strains, to enable strain identification (Fig. 4). While four samples carried some not genotypable positions and whose strain genotyping may not be accurate, sample ‘128’ was classified as ‘Alpha’ B.1.1.7 strain, samples ‘18’, ‘80’, ‘241’ and ‘326’ were classified as B.1.177 strain, sample ‘41’ was classified as B.1.36.8 strain and sample ‘123’ was classified as B.1.404 strain. All identified strain genotypes were confirmed to be circulating at the time and place of sampling, according to the GISAID database [34].

### Validation of STArS protocol on Flongle flow-cell

In order to assess the flexibility of the STArS protocol, we performed a second sequencing experiment (Run 2) on a Flongle flow-cell, that represents the lower output and cost-effective alternative to standard MinION flowcells. Clinical samples with variable viral load (Ct 25.0 and 30.0) and a clinical sample assessed as negative by RT–qPCR were sequenced in the same ONT sequencing run, together with a NTC and a PC sample (Table 2). After 15 h, a total of 5263 reads were generated. Of those, 904 reads (17.2%) were retained after filtering based on quality and length and could be demultiplexed with stringent parameters. Despite the low number of reads, STArS detected SARS-CoV-2 in two out of three clinical samples, in agreement with RT–qPCR results and confirming the reliability of the previously defined scoring rule (Table 4).

In terms of viral genotyping, sample ‘10’ was classified as B.1.1.28.7 strain, while sample ‘09’ could not be genotyped due to the low sequencing read coverage (Supplementary Fig. S1).

## Discussion

In this work, we described a workflow for the detection and genotyping of SARS-CoV-2 viral infections, which builds upon the widely adopted tiling amplicon approach developed by the ARTIC consortium [29]. Compared to the original ARTIC protocol, some simplifications have been introduced, such as a reduction in the number of quantification and purification steps, aiming for a widespread adoption of this protocol as an agile diagnostic tool. Instead of using the whole set of 98 amplicons of the ARTIC protocol, only 10 are exploited by the STARS protocol, in particular 8 covering variant sites and enabling strain discrimination and 2 covering the most frequently used genomic targets by traditional RT–qPCR assays [53]. By testing the protocol on both a MinION and a Flongle flow-cell, we showed it can easily be scaled to variable batch sizes.

Phylogenetic analyses of SARS-CoV-2 sequences deposited in the public GISAID database showed the presence of at least five clades characterized by geographic and genomic specificity and different spread periods [53, 54]. Each strain type is characterized by some co-occurring mutations, and this allows to determine strain type by sequencing only informative portions of the SARS-CoV-2 genome [53]. Since different positions along the genome are in linkage disequilibrium, strain assignment is expected to be effective also in presence of partial genomes [55]. Accordingly, our STArS approach is based on the sequencing of 10 amplicons of the ARTIC panel, that span the genomic regions where the most informative variants for SARS-CoV-2 strain identification occur [53]. Exploiting such panel, the assay can discriminate the most prevalent SARS-CoV-2 strains, namely Omicron, Delta, Alpha, Beta and Gamma. In particular, some amplicons contain mutations present in only one of those strains, as for example amplicons 93 and 95, as reported in PANGO Lineage [56]. In addition, on the N gene, variant del31/33 is attributed only to Omicron strain and variant D63G only to the Delta strain, covered respectively by amplicons 93 and 94. Moreover, we included a number of amplicons to cover more than one variant per strain (six on average), to enable genotyping even in case of possible amplicon drop-out. Beside careful amplicon selection, we ensured correct strain assignment by exploiting a software designed to work also with partial genome data, excluding missing regions from the computed metrics [57]. Despite such precautions, the STArS workflow retains the same limitations of sequencing-based approaches for SARS-CoV-2 genotyping, when analysing samples with very low viral load and/or heavy degradation [32, 58]. In these cases, it is possible that the amplicon drop out is so important that the strain is occasionally mis- or not-classified, as for a set of samples in our study.

Detection of SARS-CoV-2 presence is performed by STArS based on the number of amplicons that are sequenced, leveraging a score that takes into account the presence of signal above the negative control, in terms of number of reads mapped to SARS-CoV-2 genome. Although the scoring system to classify positive/negative patients was assessed hereby in a limited set of patients, it was adapted from Wang *et al*. [4], that validated it in a large set of samples. In our sequencing runs, we identified that a threshold of 2 could discriminate positive versus negative samples. However, positive samples with 2 ≤ score ≤ 4 had very low viral load according to RT–qPCR, namely Ct > 34. Since RT–qPCR results with such high Ct values are frequently classified as inconclusive [58], the user may want to adopt a more conservative threshold for positive classification (e.g. score >4). In case a larger number of positive and negative samples are sequenced, we envisage an improved scoring system based on logistic regression. This new scoring system may predict SARS-CoV-2 infection probability based on the strength of the signal above the negative control across the set of amplicons. The STArS protocol showed high sensitivity for the detection of SARS-CoV-2 infection, similar to the RT–qPCR approach and highly correlated to the latter. Consistently, the limit of detection was 10^7^ viral copies, comparable to the detection limit of a standard RT–qPCR assay (Ct ∼40). The introduction of a PC with a Ct close to the limit of detection makes the analysis more robust and has a threefold aim [59]. First, it allows a comparative quantification of the viral load of positive samples. Secondly, it can be exploited for determining whether more sequencing reads should be produced or not, in real time. Thirdly, it allows preventing pores from being damaged due to low pore occupancy, in case only negative samples are being sequenced in the same run [59]. On the other hand, the inclusion of a negative control allows an accurate monitoring of contamination issues and, through the application of a scoring rule, a reliable identification of positive infection can be achieved [4].

Analysis of coverage depth across all samples revealed that in high viral load samples (Ct < 30) all target regions were detected, with relatively even amplicon balance and genome coverage. For these samples, strain genotyping could be successfully achieved, thanks to combined information from multiple amplicons. Conversely, in low viral load samples (Ct > 30), some of the target regions were lost, an issue already reported in the literature for amplicon sequencing with low viral loads [26, 32]. Despite issues in precise genotyping of these samples, our STArS system succeeded in classifying all clinical samples as positive for SARS-CoV-2 infection, in agreement with RT–qPCR data.

This proof of concept study showed that the STArS protocol is not only suitable to genotype already confirmed SARS-CoV-2 viral infections, but it can also be used for viral detection and quantification, with results comparable to gold-standard assays. Although the present version of the assay was designed for monitoring the most abundant circulating strains in May 2021, we are able to discriminate also the Delta and Omicron variants. Moreover, the modularity and flexibility of STArS protocol allows the smooth substitution or inclusion of novel amplicons of interest, to better pursue current monitoring tasks and eventually to identify new variants of concern.

After the release of the ARTIC amplicon scheme, alternative protocols for SARS-CoV-2 sequencing were proposed [4, 27, 28, 60], that exploit longer amplicons (up to 2.5 kb). Although these amplicons could be directly sequenced by ONT without the need of fragmentation, reducing library prep costs, they may struggle to amplify positive samples that are even just partially degraded and/or low concentrated [29]. These features frequently characterize clinical samples and may lead to the loss of big contiguous portions of the SARS-CoV-2 genome, undesirable especially in the correspondence of critical variant sites (e.g. S gene). Moreover, they have been shown to produce a higher percentage of reads that are unsuitable for bioinformatic analysis, likely due to both fragmentation of synthesized molecules and prematurely aborted molecules during sequencing [60]. Recently, novel protocols have been proposed which are based on the sequencing of the Spike gene [31, 61]. Although these protocols share a similar underlying philosophy, STArS protocol benefits of sequencing additional genomic regions corresponding to RT–qPCR amplicons and builds upon the largely tested and iteratively improved ARTIC amplicons scheme. Finally, thanks to the use of an internal control and the absence of amplicon normalization, it provides the opportunity to obtain a quantification of SARS-CoV-2 proportional to the commonly used RT–qPCR assay, an aspect usually neglected in sequencing-based analysis, but important to assess the viral load.

In conclusion, compared to available workflows, STArS represents a compromise between presence/absence screening tests and WGS approaches, providing a 2-fold information at diagnostic aims: viral detection and strain genotyping. Considering a multiplex of up to 12 samples on a Flongle flow-cell, we estimated a cost of about US$30 per sample, very close to a RT–qPCR assay (US$10–15) but consistently lower than a WGS analysis (>US$100). The advantages of portability and reduced turnaround time of ONT sequencing platform coupled with the presented protocol will provide an important complementary tool for combatting the global pandemic that can be possibly implemented in the diagnostic settings upon appropriate clinical validation.

## Supplementary data

Supplementary data is available at *Biology Methods and Protocols* online.

## Supplementary Material

bpac020_Supplementary_Data

## Data Availability

The raw sequencing data supporting the conclusions of this article are available at the NCBI SRA repository under BioProject ID PRJNA835480.
