# Supplementary material for: STArS (STrain-Amplicon-Seq), a targeted nanopore sequencing workflow for SARS-CoV-2 diagnostics and genotyping
Source: Biol Methods Protoc. 2022 Aug 25;7(1):bpac020. doi: 10.1093/biomethods/bpac020 (PMC9422081; doi:10.1093/biomethods/bpac020)
Supplement: bpac020_Supplementary_Data [file bpac020_supplementary_data.pdf]

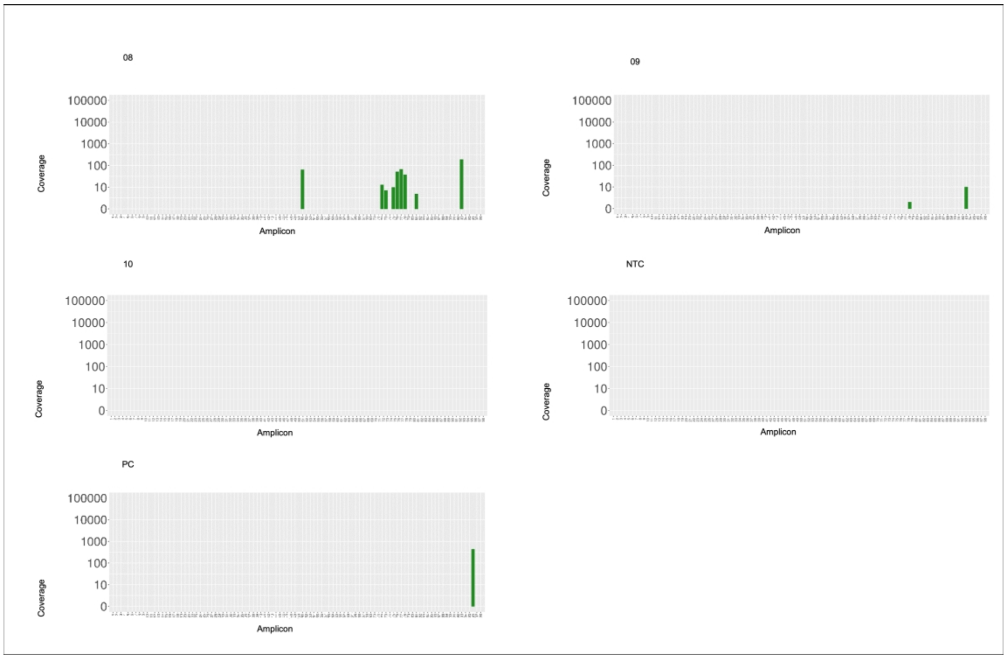

Figure S1. SARS-CoV-2 genome sequencing coverage for Run 2. For each sample, the number of reads mapped to each amplicon is reported in log10 scale.

716x465mm (130 x 130 DPI)
